# Ramadan Fasting in Metabolic Dysfunction-Associated Steatotic Liver Disease: Linking the Gut–Mitophagy–Circadian Axis to Endothelial Dysfunction and Hepatic Outcomes

**DOI:** 10.3390/jpm16090474

**Published:** 2026-09-15

**Authors:** Musaab Ahmed, Nisha Shantakumari, Safaa Badi, Mohamed H. Ahmed

**Affiliations:** 1 College of Medicine, Ajman University, Ajman P.O. Box 346, United Arab Emirates; n.kumari@ajman.ac.ae; 2 Department of Clinical Pharmacy, Faculty of Pharmacy, Omdurman Islamic University, Khartoum 14415, Sudan; safaabadi30@gmail.com; 3 Department of Medicine for Older People and Department of Diabetes, North Cheshire and Mersey NHS Foundation Trust, Warrington WA5 1QG, UK; mohamed.hassan-ahmed@nhs.net; 4 Faculty of Medicine and Health Sciences, University of Buckingham, Buckingham MK18 1EG, UK

**Keywords:** metabolic dysfunction-associated steatotic liver disease, Ramadan fasting, endothelial dysfunction, gut microbiota

## Abstract

Metabolic dysfunction-associated steatotic liver disease (MASLD) is the predominant etiology of chronic liver disease and the leading factor to liver-related mortality. The endothelial dysfunction is a significant contributor to the onset of cardiovascular disease and is closely linked to MASLD. Previous studies have shown that changes in diet influence both MASLD and endothelial function and modify their risk factors. A small number of studies have evaluated the possible effects of Ramadan fasting on MASLD, related endothelial dysfunction and the gut–mitophagy–circadian axis. The aim of this narrative review is to evaluate the impacts of Ramadan fasting on MASLD, hepatic complications, related endothelial dysfunction and the gut–mitophagy–circadian axis. This review integrates endothelial dysfunction, gut microbiota, mitophagy, and circadian rhythm into a single mechanistic framework. We performed searches of SCOPUS and the PubMed databases using diverse search keywords. The review targeted scholarly English articles from the year 2000 to 2026. The review included seventy-three studies. Ramadan fasting might enhance MASLD, hepatic complications, and associated endothelium dysfunction and alter risk factors by facilitating weight loss, ameliorating insulin resistance, enhancing mitophagy, and inducing alterations in gut microbiota and circadian gene expression. Further studies are necessary to assess safety and efficacy of Ramadan fasting on MASLD, hepatic complications and related endothelial function. Circadian control offers a significant molecular foundation connecting Ramadan fasting to metabolic health.

## 1. Introduction

Metabolic dysfunction-associated steatotic liver disease (MASLD) is the predominant etiology of chronic liver disease [1]. It presents a considerable worldwide health problem owing to its rising frequency and robust correlation with cardiovascular disease [2]. Cardiovascular disease in MASLD commonly progresses gradually, with pathological changes often occurring years before the emerging of the clinical manifestations [3]. Endothelial dysfunction is characterized by variations in endothelial functions, including various maladaptive changes in the functional phenotype that affect the regulation of inflammation, thrombosis, hemostasis and vascular tone [4]. Cardiometabolic risk factors, including hypertension, abdominal obesity, dyslipidemia, and insulin resistance, are strongly associated with endothelial dysfunction [5]. Importantly, heart failure, diabetes and MASLD are recognized risk factors of endothelial dysfunction [6]. Mechanisms responsible for endothelial dysfunction in MASLD include inflammation, oxidative stress and alteration in lipoprotein metabolism [2]. Gut microbiota contributes to MASLD and endothelial dysfunction [7,8]. Circadian rhythms are internal biological cycles with an approximate 24-hour duration that govern several physiological and metabolic functions, including sleep–wake patterns, hormone release, glucose regulation, lipid metabolism, immunological response, and energy equilibrium. The central circadian clock in the hypothalamus regulates these rhythms, synchronizing peripheral clocks in the liver, pancreas, adipose tissue, skeletal muscle, and gastrointestinal tract. Circadian rhythms at the molecular level are governed by interrelated transcriptional–translational feedback loops that include the core clock genes CLOCK, CRY1–2, BMAL1, and PER1–3 which modulate the rhythmic expression of genes associated with inflammation, metabolism, oxidative stress, mitochondrial function, and autophagy [9,10]. Ramadan fasting has several benefits, including reductions in body weight and composition, along with enhancements in aspects of metabolic syndrome, including insulin resistance. Furthermore, fasting throughout Ramadan enhances plasma glucose levels and diminishes inflammatory markers and oxidative stress [11]. Although emerging evidence links gut microbiota, mitochondrial quality control, and circadian regulation with metabolic dysfunction and MASLD, the extent to which these pathways interact during Ramadan fasting remains incompletely established. In particular, direct human evidence demonstrating a coordinated gut–mitophagy–circadian pathway in MASLD during Ramadan is currently limited. Therefore, the gut–mitophagy–circadian axis discussed in this review should be regarded as a proposed mechanistic framework that integrates findings from human Ramadan studies with evidence from experimental and mechanistic studies of fasting, metabolism, mitochondrial quality control, and circadian biology. This distinction is important because improvements observed during Ramadan may also reflect changes in energy intake, meal composition and timing, sleep–wake patterns, physical activity, and other behavioral factors. This review therefore discusses the pathophysiology of MASLD and endothelial dysfunction and examines the potential effects of Ramadan fasting on MASLD and its hepatic complications, endothelial function, gut microbiota, mitophagy, and circadian regulation. Particular emphasis is placed on the potential interactions among these systems while distinguishing established clinical observations from mechanistic hypotheses.

## 2. Methods

**Figure 1:**
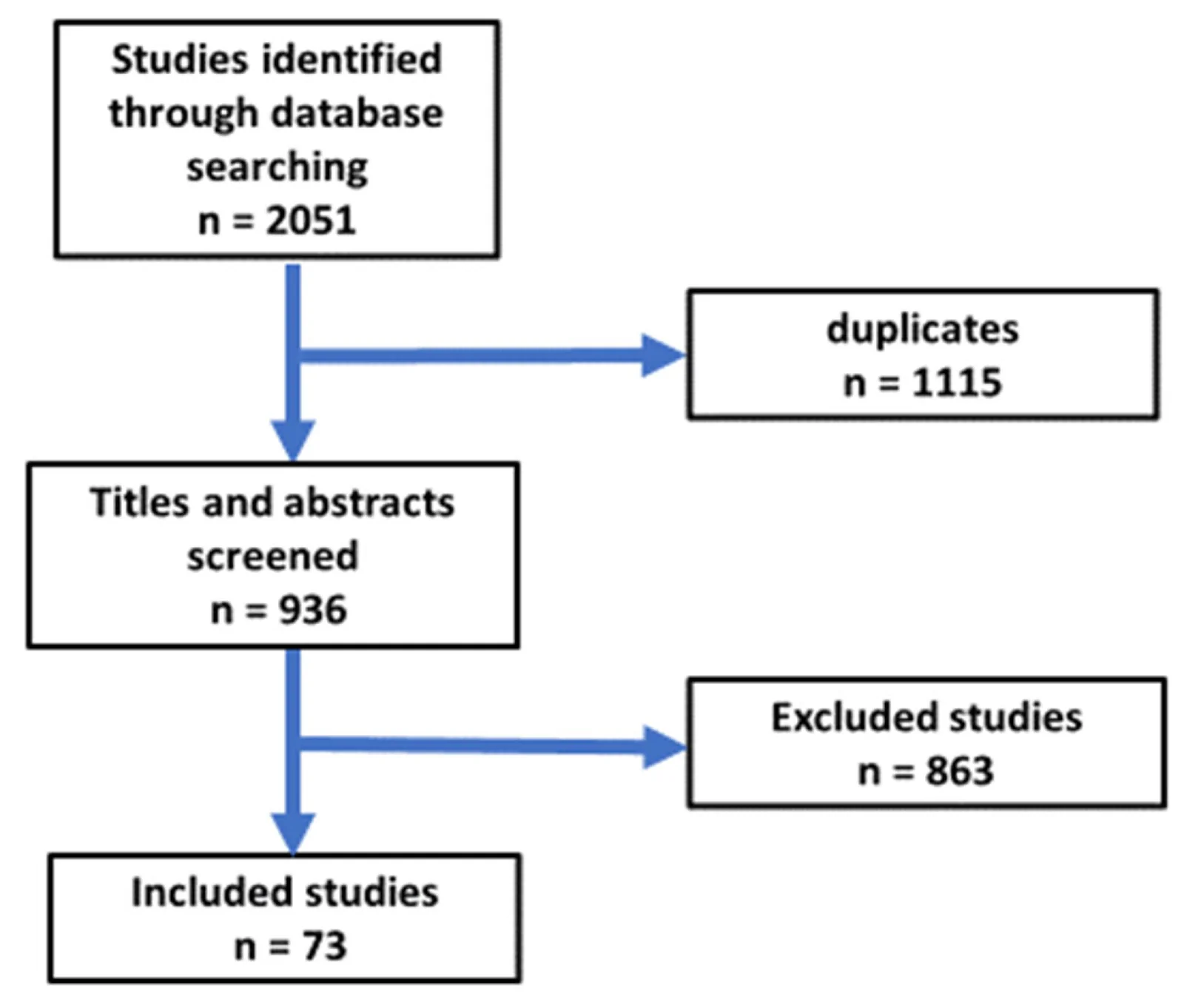
Flow chart for the selection of publications.

This narrative review was conducted to synthesize the available evidence regarding Ramadan fasting, metabolic dysfunction-associated steatotic liver disease (MASLD), endothelial dysfunction, gut microbiota, autophagy/mitophagy, circadian biology, and hepatic complications. This narrative review included the searching of SCOPUS and the PubMed databases using the words: MASLD, gut microbiota, endothelial dysfunction, liver cirrhosis, hepatocellular cancer, oxidative stress, and Ramadan fasting. The authors conducted a search with a conjunction of the following terms: [Ramadan Fasting AND MASLD OR endothelial dysfunction OR Gut microbiota OR liver cirrhosis OR hepatocellular carcinoma OR Oxidative stress OR mitophagy OR circadian rhythm AND clinical trials]. The assessment of the literature targeted research articles in English from 1 January 2000 to 31 March 2026. The abstracts and articles were evaluated. Articles were thoroughly analyzed, and data regarding Ramadan intermittent fasting, participant numbers, duration of study, and impacts on MASLD, endothelial dysfunction, gut microbiota, liver cirrhosis, hepatocellular carcinoma, oxidative stress, mitophagy and circadian rhythm were extracted. The list of references contained relevant citations. First search produced a total of 2051 items. A total of 73 articles were selected for this study (Figure 1).

Inclusion Criteria: Only randomized controlled trials, review, observational studies, and experimental research were included into this review article for the discussion.

Exclusion criteria: Letters to the editor, comments, news articles, case reports, books, notes, theses, brief surveys, duplicate studies, conference abstracts, publications not in English, duplicates, studies unrelated to the review objectives, lacked sufficient information to interpret the relevant outcome, or addressed fasting paradigms with no meaningful relevance to the specific mechanistic or clinical question under consideration were excluded.

All gathered documents were imported into EndNote for the removal of duplicates. Following de-duplication, records were screened in two successive stages. First, titles and abstracts were assessed for relevance according to the eligibility criteria. Articles deemed relevant were then retrieved in full text and assessed independently by two reviewers. Discrepancies in inclusion decisions were resolved through discussion or in consultation with a third senior reviewer where needed.

## 3. Metabolic Dysfunction-Associated Steatotic Liver Disease

MASLD is an intensifying worldwide pandemic. The MASLD pandemic is a significant public health concern [12]. The accumulation of triglycerides and free fatty acids in the liver is mostly attributed to insulin resistance and obesity. Individuals confirmed to have MASLD have an increased susceptibility to mortality due to hepatic and cardiovascular diseases. MASLD is a serious criterion for liver transplant [13].

MASLD is a designation that contains several diseases, ranging from hepatic fat deposition to steatosis with hepatitis, cirrhosis, fibrosis, and hepatocellular carcinoma, all manifesting without considerable use of alcohol. MASLD encompasses two principal disorders: non-alcoholic fatty liver (NAFL) and metabolic dysfunction-associated steatohepatitis (MASH) [14]. Non-alcoholic fatty liver is defined by the buildup of hepatic fat exceeding 5 percent of the liver’s tissue, without causing cellular injury. MASH is a disorder manifested by inflammation and death of hepatocytes, along with the accumulation of fat (steatosis) [15].

Most individuals with MASLD exhibit insulin resistance and obesity which significantly facilitates the occurrence of metabolic syndrome [16,17]. MASLD is the liver-related expression of metabolic syndrome, defined by a combination of disorders such as hypertension, central obesity, hyperglycemia, hypertriglyceridemia, and reduced high-density lipoprotein levels. These irregularities raise the risk of cardiovascular ailments, cerebrovascular incidents, and type 2 diabetes [18].

## 4. Pathogenesis of MASLD

The pathogenic mechanism of MASLD is not completely comprehended. The principal feature of the syndrome is the accumulation of triglycerides and free fatty acids, primarily attributed to obesity and insulin resistance [19].

**Figure 2:**
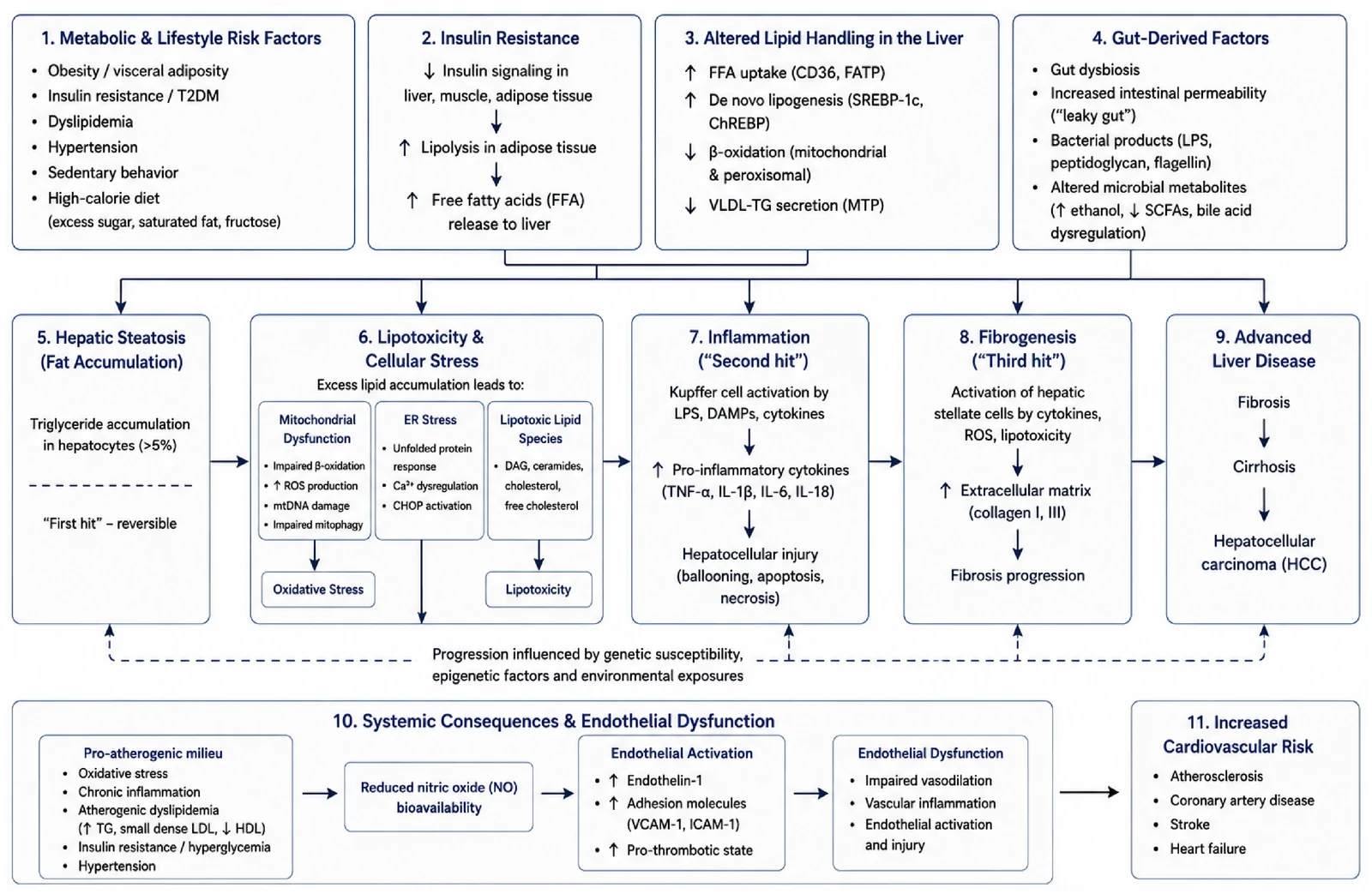
Pathogenesis of MASLD.

The pathogenesis of MASLD was elucidated by the two-hit model. The initial hit includes the factors contributing to hepatic steatosis, such as a high-fat diet, lack of physical activity, accumulation of hepatic lipids, obesity, and insulin resistance [20]. The second hit pertains to the factors that incite cellular death, inflammation, and fibrosis, including oxidative stress, proinflammatory cytokines, endoplasmic reticulum stress, and gut-derived bacterial endotoxins [21]. The two-hit model was considered inadequate in thoroughly elucidating the progression of MASLD, as multiple factors operate concurrently in an individual with genetic predisposition [20]. This corroborates the assertion of a multi-hit model in 2010 [22]. Insulin resistance has a substantial function in the occurrence of oxidative stress, proinflammatory cytokines, endoplasmic reticulum stress, and bacterial endotoxins originating from the gut. Hepatic steatosis results in hepatic de novo lipogenesis, a decrease in lipolysis within adipose tissue, and an elevation of fatty acids in the liver [23]. Furthermore, insulin resistance induces alterations in the production and secretion of adipokines and inflammatory cytokines [24]. The accumulation of liver triglycerides results in heightened production of reactive oxygen species, causing mitochondrial impairment and endoplasmic reticulum stress [25]. The gut microbiota is believed to play an important role in the progression of MASLD. The gut microbiota impacts nutritional absorption and excretion in the liver. This takes place due to their provision of toll-like receptor ligands, which activate the liver to generate more proinflammatory cytokines. Consequently, probiotics have been suggested as a possible remedy for MASH by modifying the gut microbiota makeup [26]. The characteristics following liver transplantation encompass the traits of metabolic syndrome and MASLD representing the hepatic manifestation of this disorder. Consequently, it is common for individuals to experience a recurrence or the emergence of new instances of MASLD or MASH post-liver transplantation [27]. The incidence of MASLD post-liver transplantation varies between 18% and 40%. Variations in PNPLA3, which aid in the degradation of triglycerides, is related with a heightened risk of developing MASLD post-liver donation. These polymorphisms are additionally associated with pre-transplant obesity and MASLD. The emergence of MASLD post-liver transplantation may plausibly exacerbate the increased cardiovascular mortality observed in these patients [28] (Figure 2).

### 4.1. Pathophysiology of Endothelial Dysfunction

The endothelial dysfunction pathophysiology is intricate and has various mechanisms. The oxidative stress takes part in pathogenesis of endothelial impairment. It arises from various enzymatic pathways, including xanthine oxidase, uncoupled endothelial nitric oxide synthase (eNOS), NADPH oxidases, and impaired mitochondria, occurring when the equilibrium between antioxidant and pro-oxidant systems is disrupted [29]. Increased concentrations of reactive oxygen species (ROS) oxidize intracellular macromolecules, which in turn diminishes nitric oxide (NO) synthesis via peroxynitrite formation, resulting in the eNOS cofactor tetrahydrobiopterin degradation, thereby uncoupling eNOS and enhancing pro-oxidant activity. Oxidative stress correlates with reduced endothelial vasodilation and an inflammatory state. Furthermore, it enhances the expression of adhesion molecules, including VCAM-1 and ICAM-1, along with chemotactic molecules [30]. Inflammation plays a crucial role in the development of cardiovascular disease [31]. Endothelial cells generate a range of substances in reaction to damage, such as interleukin-8, chemokines, interferons, monocyte chemoattractant protein-1, colony-stimulating factors, intercellular adhesion molecule-1, P-selectin, E-selectin, growth factors, vascular adhesion molecule-1, and additional inflammatory agents [29]. This leads to improved adhesion and movement of leukocytes across the endothelium, resulting in the activation of a proinflammatory condition [32,33]. Moreover, proinflammatory mediators act to stimulate the endothelial cells directly to secrete additional proinflammatory cytokines, thus sustaining a detrimental cycle [34]. This fluctuation results in a prothrombotic, proliferative, and proinflammatory state manifestation of impaired endothelial cells.

### 4.2. Mechanisms of Endothelial Dysfunction in MASLD

Endothelial dysfunction takes parts in the onset of cardiovascular disease and is related to MASLD. The endothelium maintains vascular equilibrium through regulation of blood flow, coagulation, and vascular tone. It often produces factors that facilitate vasodilation and suppress platelet aggregation and smooth muscle cell proliferation [35]. MASLD disturbs this equilibrium, producing endothelial impairment and a heightened likelihood of cardiovascular incidents [36]. Nitric oxide, a vital endothelium-derived relaxant, is imperative for endothelial well-being, promotion of vasodilation and inhibition of aggregation of platelets and smooth muscle cell growth [37]. In MASLD, the production of nitric oxide is compromised due to heightened oxidative stress and inflammation, leading to diminished nitric oxide levels. Asymmetric dimethylarginine (ADMA), a natural antagonist of endothelial nitric oxide synthase (eNOS), significantly plays a major role in endothelial impairment in individuals with MASLD and cardiovascular disease (CVD). Elevated ADMA concentrations, in MASLD, hinder eNOS activity and hence diminish nitric oxide. The reduction in nitric oxide leads to increased vasoconstriction, reduced vasodilation, and heightened vascular resistance and blood pressure. Increase in ADMA in MASLD is related to elevated methylation in liver, coupled with extensive oxidative stress and inflammation, which also hampers nitric oxide production and intensifies endothelium-dependent vasodilation [38,39]. This repression results in heighted arterial rigidity and a higher likelihood of thrombosis, reducing the role of ADMA as a facilitator of vascular impairment in MASLD and the correlated rise in cardiovascular disease risk. Furthermore, endothelin-1 (ET-1), is overproduced in MASLD due to oxidative and proinflammatory conditions. ET-1 increased synthesis in MASLD leads to detrimental vasoconstriction and vascular smooth muscle proliferation, endothelial dysfunction and atherosclerosis [40].

## 5. Oxidative Stress

Oxidative stress occurs due to a disparity between the generation of reactive oxygen species (ROS) and antioxidant mechanisms. It is integrally associated with the pathophysiology of MASLD and the likelihood of CVD [41]. In MASLD, hepatic fat storage elevates ROS generation, resulting in cellular damage and fostering inflammation and fibrosis. This may exacerbate insulin resistance and amplify metabolic dysfunction [42]. In the context of cardiovascular disease, oxidative stress exacerbates atherosclerosis by impairing the endothelium, triggering inflammation, and oxidizing fats, ultimately leading to arterial plaque formation [43,44]. Elevated OS might adversely affect cardiovascular function by damaging cardiac myocytes and blood arteries, potentially resulting in myocardial infarction, hypertension, and heart failure [45]. Moreover, obesity, present in both MASLD and CVD, induces oxidative stress by the generation of nutrients that elevate LDL and VLDL levels and facilitate lipid peroxidation, consequently heightening the probability of CVD [46].

## 6. Inflammation

Systemic inflammation has a pivotal role in the development of MASLD [41]. Inflammation transcends the liver, profoundly affecting the heart and blood vessels [47]. The release of inflammatory cytokines and chemokines is increased in MASLD, most notably TNF-α and IL-6, which play crucial roles as regulators. Research in mice has shown that tumor necrosis factor α (TNFα) plays an important role in regulating inflammation and insulin resistance in the liver, which are associated with the development of MASLD in non-obese individuals, and crucial for the progression of MASLD in obese individuals [48].

Simultaneously, liver inflammation and fatty liver disease are the main factors associated with TNF-α production. The overproduction of numerous proinflammatory mediators is facilitated by TNF-α through its activation of the nuclear factor kappa-B (NF-κB) signaling pathway. In addition, TNF-α is a key player in the development of atherosclerosis because it promotes monocytes to transform into foam cells [49]. The IL-6 signaling cascade activates acute-phase proteins such as C-reactive protein (CRP) and serum amyloid A (SAA), leading to transcriptional upregulation. This, in turn, enhances the inflammatory response in the liver and has systemic consequences outside of the liver. Atherosclerosis progresses due in large part to persistent low-grade inflammation, which is a feature of MASLD. Plaque buildup inside the arteries is the hallmark of atherosclerosis. Excessive levels of proinflammatory cytokines like TNF-α and IL-6 can worsen MASLD by causing endothelial dysfunction, unstable plaque, and damage to the arterial wall. This, in turn, can aid in the progression of atherosclerosis and coronary artery events [50].

## 7. Alteration in Lipoprotein Metabolism

Atherosclerosis and cardiovascular disease (CVD) is greatly exacerbated in MASLD by dyslipidemia. An important step in the formation of atherosclerotic plaques is the induction of inflammatory processes by small dense low-density lipoprotein (sdLDL) particles, which are able to cross the endothelium and convert into ox-LDL. Because they have lesser affinity for LDL receptors, their atherogenic action is amplified by their slower clearance [51]. Reduced levels of HDL also reduce the antioxidant and anti-inflammatory effects of HDL, which makes it harder for the body to break down excess cholesterol, and impede reverse cholesterol transit. A pro-atherosclerotic lipid composition is promoted by the overproduction of VLDL and reduced HDL activity due to inadequate lipid management in the liver [52]. The macrophages absorption of sdLDL results in foam cell production, worsening endothelial dysfunction and oxidative stress, hence accelerating the course of atherosclerosis [2].

## 8. Endothelial Dysfunction and Complications of MASLD

### 8.1. Endothelial Dysfunction and Liver Cirrhosis

Cirrhosis causes an increase in intrahepatic vascular resistance due to the induction of capillarization and a decrease in nitric oxide (NO) generation due to oxidative stress and overexpression of GRK2 [53].

The hypercoagulable condition and endothelial dysfunction associated with cirrhosis are greatly influenced by platelet-derived TGF-β1. There was a considerable correlation between TGF-β1 concentrations and both platelet count (r = 0.733; *p* < 0.001) and thromboelastography parameters, and TGF-β1 levels were noticeably higher in patients with PVT. The production of vWF, thrombomodulin, ICAM-1, and VEFG was enhanced when liver sinusoidal endothelial cells were stimulated in vitro by TGF-β1, which lends credence to its own prothrombotic and endothelium-damaging actions [54]. Additionally, the activation of systemic coagulation is enhanced by the production of prothrombotic microvesicles, especially those originating from endothelial cells, platelets, and leukocytes that exhibit tissue factor [55].

The risk of portal vein thrombosis (PVT) increases due to endothelial impairment and persistent inflammation caused by portal hypertension, which in turn leads to vascular injury. The portal circulation is diminished in hepatocellular carcinoma patients with portal vein tumor thrombosis (PVTT) as a result of direct vascular infiltration; the flow velocity decreases as the thrombus load increases. Portal hypertension and hepatic perfusion are both exacerbated by the possibility of a complete blockage of flow due to the major portal trunk’s engagement. Doppler ultrasonography frequently shows reduced or different flow patterns in such a case. Under normal physiological conditions, endothelin 1 (ET1) boosts vasodilatory Akt/eNOS signaling via ET B and controls sinusoidal tone through stimulating stellate cell contraction through ET A receptors. Phosphorylation of eNOS by PI3K enhances NO generation through activation of vascular endothelial growth factor receptor 2 (VEGFR 2), which is independent of VEGFA alone. This vasodilatory effect is enhanced and platelet aggregation is inhibited by prostacyclin (PGI2). Cirrhosis causes intrahepatic vasoconstriction because oxidative stress lowers prostacyclin and nitric oxide availability. The absence of microRNA 126 disrupts angiogenic equilibrium and endothelial healing, while angiopoietin 2 (Ang 2) weakens sinusoidal vessels’ stability and makes the endothelium more susceptible to inflammatory damage. A pro-thrombotic condition is promoted when the endothelial protein C receptor (EPCR) and thrombomodulin (TM) are both downregulated at the same time. This affects the protein C anticoagulant pathway [56].

### 8.2. Endothelial Dysfunction and Hepatocellular Carcinoma

The release of IL-6, TNF-α, and VEFG, along with the expression of TF, worsen endothelial dysfunction in HCC. TF activates endothelial protease-activated receptors (PARs), which in turn induce localized coagulation [57]. This prothrombotic environment is influenced by epigenetic changes; for instance, hypomethylation of cancer-causing genes, such as FAM83D and TNFRSF10A, improves tumor cell motility and decreases endothelium stability [56,58]. However, the apoptotic clearance of cancer clones is hindered by hypermethylation of tumor suppressor genes, notably SCAND3 [59]. Long non-coding RNAs (lncRNAs) that interact with CDK5, FOSL2, and CD44v6 boost endothelial mesenchymal transition, matrix reorganization, and immune modulation; repression of miR 381 enhances VEGFA expression, which promotes vascular permeability and angiogenesis [60,61].

### 8.3. Gut Microbiota and MASLD

A study conducted by Zhang et al. showed that time-restricted feeding significantly improved the gut microbiota, particularly Ruminococcus torques, while the microbial metabolite 2-hydroxy-4-methylpentanoic acid suppressed the intestinal pathway of Hypoxia-inducible factor-2alpha sphingolipid, thereby mitigating inflammation and fibrosis in MASH [62]. Sun et al. found that short-chain fatty acids possess anti-inflammatory effects through obstructing the NF-κB signaling pathway, reducing the synthesis of inflammatory cytokines, and mitigate inflammation of the liver in MASLD [63]. Additionally, Liu et al. indicated that short-chain fatty acids strengthen gut barrier integrity, reduce bacterial translocation and endotoxin infiltration, hence mitigating hepatic inflammation [64].

### 8.4. Gut Microbiota and Endothelial Dysfunction

Endothelial dysfunction is characterized by additional alterations, including endothelial-to-mesenchymal transition and increased cell apoptosis resulting in vascular leakage, inflammation, and coagulation [65]. The metabolites produced by the gut microbiota have diverse impacts on endothelial function. The intestinal microbiota can impact the endothelium of the circulatory system through two main pathways: first, the microbiota and its byproducts might stimulate the enteric nervous system, influencing brain regions that govern cardiovascular regulation; second, they can permeate the blood–intestinal barrier, altering the functionality of tissues responsible for circulatory equilibrium. Thus, preserving the microbial balance in a state of eubiosis and alleviating intestinal dysbiosis is proposed as a strategy to reduce endothelial and vascular impairment [7].

### 8.5. Gut Microbiota and Liver Cirrhosis

Dysbiosis of the gut microbiota is significantly correlated with cirrhosis. Research on microbiota composition revealed notable variability in the findings. Nonetheless, alterations in alpha diversity, particularly the proliferation of pathobionts such as Enterococcus and Streptococcus, alongside the reduction in species from bacterial families crucial for homeostasis of the gut, encompassing Lachnospiracaceae and Oscillospiracaceae, were associated with the advancement of liver cirrhosis. The alterations in composition of microbiota directly and indirectly influence the pathophysiology of the cirrhosis by multiple pathways, chronic inflammation, metabolic abnormalities, and heightened intestinal permeability [66].

### 8.6. Gut Microbiota and Hepatocellular Carcinoma

Alterations in gut microbiota are observed not only in hepatocellular carcinoma but also in patients with liver disorders of diverse origins. The characteristics of these alterations differ according to the root reasons, and the composition of gut microbiota generally fluctuates about the bacteria involved in each case. Thus, it is advised that the examination of gut microbiota could aid in pinpointing a particular trigger of HCC to support a tentative etiological diagnosis, and that it might also be done at an initial stage. Certain metabolites generated by particular intestinal microorganisms, chiefly bacteria, might be seen in peripheral blood and/or feces, serving as preliminary indicators for hepatocellular cancer [67]. Research conducted by Jinato et al. demonstrated α-diversity of IM was significantly reduced in NBNC-HCC relative to healthy controls and viral HCC. Sixteen bacterial genera were recognized that displayed notable disparities between NBNC-HCC and viral HCC; eleven genera were predominant in viral HCC (such as Faecalibacterium, Agathobacter, and Coprococcus), whereas five genera were heightened in NBNC-HCC (including Parabacteroides, Bacteroides, Ruminococcus gnavus group, Streptococcus, and Erysipelatoclostridium). The concentrations of the fecal BCoAT gene and other fecal SCFA-producing bacteria were notably decreased in NBNC-HCC relative to viral HCC and control groups. The concentrations of lipopolysaccharide-binding protein in plasma were heightened in NBNC-CHC relative to viral HCC and control cohorts. This research discerned 16 unique genera that distinguish viral HCC from NBNC-HCC [68].

### 8.7. Gut Microbiota and Mitophagy

Short-chain fatty acids (SCFAs), especially butyrate, are recognized for their ability to promote mitophagy by enhancing genes related to mitochondrial biogenesis and autophagy primarily via the Peroxisome Proliferator-activated Receptor Gamma Coactivator 1 Alpha (PGC-1α)–Silent Information Regulator 1 (SIRT1) signaling pathway [69]. SCFAs also assist in maintaining the equilibrium of NAD+/NADH concentrations, which are crucial for the activation of sirtuins, therefore regulating mitochondrial turnover and lifespan. Conversely, gut dysbiosis results in excessive LPS synthesis, an endotoxin that impairs mitophagy through activation of the NOD-like receptor. This elevates mitochondrial reactive oxygen species (ROS), impairs mitochondrial DNA, and reduces mitophagic efficacy [70,71].

### 8.8. Ramadan Fasting

Ramadan fasting is a spiritual practice for Muslims, taking place over a month every lunar cycle [72]. Ramadan intermittent fasting is a special kind of intermittent fasting wherein Muslims abstain from consumption of food or beverages, smoking, or receiving medication from sunset until dawn for the duration of Ramadan [73,74]. Observing fast during Ramadan is spiritually required for all physically capable adult Muslims, with the length of the daily fast differing by country and fluctuating each year (spanning from 8 to 16 hours) [74]. The majority of those partaking in the Ramadan month partake in two meals each day: Suhoor, consumed prior to dawn to commence the fast, and the Iftar, savored post-sunset to end the fasting period [75,76]. This would require careful thought for patients with type 2 diabetes who intend to fast to avoid risks such as hyperglycemia, dehydration, or hypoglycemia [74,77]. Ramadan fasting yields several health benefits, such as weight reduction, diminished inflammatory indicators and oxidative stress, lowered blood pressure, and enhanced serum glucose levels [11,78].

### 8.9. Ramadan Fasting and MASLD

The available literature should be interpreted according to the degree to which it directly addresses Ramadan fasting in MASLD. Evidence specifically obtained from patients with MASLD during Ramadan remains limited. A larger body of evidence has examined Ramadan fasting in healthy individuals or in populations with obesity, diabetes, hypertension, metabolic syndrome, or other cardiometabolic abnormalities. Although these studies provide useful information regarding changes in body weight, insulin sensitivity, lipid metabolism, inflammation, oxidative stress, and vascular function, their findings cannot be assumed to represent direct evidence of benefit in MASLD. Similarly, evidence from intermittent fasting, time-restricted feeding, prolonged fasting, animal experiments, and cellular models provides mechanistic support for selected pathways but does not establish that the same responses occur during Ramadan fasting in patients with MASLD. Accordingly, throughout this review, direct clinical observations are distinguished from indirect or mechanistic evidence, and conclusions regarding hepatic protection or disease modification are presented as hypotheses requiring prospective validation.

Research on the impact of Ramadan fasting on patients with MASLD is limited (Table 1). An effective dietary therapy for MASLD could include fasting throughout Ramadan. Ramadan fasting as a dietary strategy for MASLD needs to be validated by rigorous clinical trials.

### 8.10. Ramadan Fasting and Endothelial Dysfunction

Five studies evaluated the influences of fasting during Ramadan on endothelial dysfunction. A research project carried out in Iran included twenty-one individuals suffering from cardiovascular disease and was the first investigation assessing the effects of Ramadan fasting on endothelial dysfunction. The research indicated that nitric oxide concentrations were significantly increased in patients after Ramadan fasting. Following Ramadan, levels of asymmetric dimethylarginine (ADMA) significantly diminished. Moreover, the concentrations of vascular endothelial growth factor (VEGF) increased, whereas malondialdehyde (MDA) levels decreased during Ramadan fasting; yet, these alterations were not statistically significant [84]. A separate investigation was conducted in Iran among fasting people with and without diabetes. Blood specimens were collected four weeks prior to Ramadan and two weeks following its conclusion. The research revealed a notable decrease in ICAM-1 levels across both cohorts [85]. A further investigation was conducted in Turkey in 64 hypertensive individuals who observed the fast for the entire month. Flow-mediated dilatation and biochemical metrics were assessed three days before to and following the month of Ramadan. The research indicated a notable improvement in flow-mediated dilation after Ramadan, likely due to decreased CRP levels and cortisol subsequent to the fasting duration [86]. A retrospective analysis of 67 patients exhibiting slow coronary flow utilizing TIMI frame count measurements prior to Ramadan month and one to three months post-Ramadan suggested that fasting during Ramadan and related lifestyle changes may enhance endothelial function [87]. Ahmed et al. in a recent review suggested that Ramadan fasting could enhance endothelial dysfunction and alter risk factors through facilitating weight loss and ameliorating insulin resistance. Furthermore, fasting throughout Ramadan may enhance the bioavailability and concentrations of nitric oxide [35]. Additional research is required to assess the efficacy of fasting during Ramadan on endothelial dysfunction.

### 8.11. Ramadan Fasting and Liver Cirrhosis

Limited research has investigated the effects of Ramadan fasting on liver cirrhosis. Mohamed et al. in Egypt encompassed forty individuals with liver cirrhosis, showing that cirrhotic patients had notable immediate fluctuations in portal blood circulation. Seven patients developed problems, which included two cases of variceal hemorrhage. Individuals with Child class C should not fast [88]. An observational comparative study conducted by Elnadry et al. in Egypt with 202 individuals with chronic liver disease demonstrated that fasting patients with cirrhosis had a notable reduction in Child class C after the conclusion of Ramadan [89]. In the LORANS trial and meta-analysis, Al-Jafar et al. found that cirrhotic individuals who fasted throughout Ramadan had smaller waist circumferences and hip circumferences than those who did not. It all starts in the second week of Ramadan and gradually decreases after three weeks [90]. Mohamed et al. conducted an observational study with 72 participants to examine how fasting during Ramadan affected portal hemodynamics and hepatic functions in people with liver cirrhosis. Regardless of the fasting condition, the results showed that individuals with liver cirrhosis altered portal hemodynamics and liver functionality. Portal vein congestion index Serum albumin levels and MELD score fluctuated significantly compared to healthy subjects [91].

Emara et al. in their review indicated that individuals with Child A cirrhosis can partake in Ramadan fasting, particularly if they adhere to NAFLD guidelines, contingent upon prior evaluations and diligent supervision during Ramadan. Individuals with cirrhosis classified as Child B and C ought to refrain from fasting. The likelihood of decompensation is elevated [92]. In a different review, Emara et al. showed that patients with liver cirrhosis may have higher plasma bilirubin levels during Ramadan, as well as hepatic encephalopathy, sudden gastrointestinal hemorrhage, and increased ascitic fluid production. Numerous deaths were linked to fasting throughout Ramadan, and the incidence of these results was higher in individuals with Child class B and C cirrhosis [72]. Individuals with cirrhosis classed as Child B and C should refrain from fasting, but those in the Child A category might fast after following certain measures.

### 8.12. Ramadan Fasting and Hepatocellular Carcinoma

A study by Re O et al. showed that intermittent fasting reduced the proliferation and activation of hepatic stellate cells (HSC) and amplified the anticancer efficacy of sorafenib by diminishing tumor proliferation, regulating gene expression and decreasing glucose uptake [93]. A review by Emara et al. indicated that intermittent fasting may affect the development of hepatocellular carcinoma through metabolic, inflammatory, and circadian mechanisms [72]. The suggested mechanisms that may explain the proposed antitumor effects of fasting in hepatocellular carcinoma (HCC) could involve reduction in the inflammatory response, improvement of gut microbiota, diminished insulin signaling, heightened metabolic stress in cancer cells due to decreased glucose availability and ketogenesis and upregulation of tumor suppressor genes [94]. There is currently insufficient clinical evidence to recommend Ramadan fasting as an antitumor intervention. Similarly, improvements in body weight, ALT/AST levels, lipid profile, insulin resistance, or ultrasonographic parameters should not be equated with evidence that fasting prevents fibrosis progression, cirrhosis, HCC, or liver-related clinical events. Further studies should be conducted with a high sample size to evaluate the efficacy of Ramadan fasting in the management of hepatocellular carcinoma.

### 8.13. Ramadan Fasting and Gut Microbiota

Ten studies showed the impact of Ramadan fasting on gut microbiota. Gul et al. [95] in Pakistan demonstrated that Ramadan fasting enhanced the taxonomic and functional variety of bacteria while reducing the prevalence of several detrimental microorganisms, including Blautia, Desulfovibrio, Lachnoclostridium, Haemophilus, and Porphyromonas. Intermittent fasting showed an increased prevalence of Lactobacillus, Prevotella, and Anaerostipes. Ramadan fasting resulted in a notable elevation in SCFAs such as C7, iC4, and iC6, which corresponded to variations in microbial composition and phylogeny, respectively [95]. Ozkul et al. [96] included nine subjects. The study involved seventeen hours of daily fasting from dawn to dusk over a span of twenty-nine days. Stool specimens were gathered the day before Ramadan began and on the day it ended, omitting any intake of meals heavy in glucose or fat. The research indicated that intermittent fasting during Ramadan results in a rise in A. muciniphila and the B. fragilis group, both regarded as advantageous elements of gut microbiota. Mohammadzadeh et al. recruited 30 people for their Iranian study. Before and after Ramadan, participants kept meal diaries for three days to assess their food intake. Serum butyrate levels increased significantly throughout Ramadan. After Ramadan, compared to levels before Ramadan, the intestinal Bacteroides showed a 21% rise and the firmicutes a 13% increase (*p* < 0.05) [97]. In South Korea, Jo et al. 2023 [98] studied 20 Muslims over the course of four weeks during Ramadan and eight weeks after the holy month to determine the makeup of their gut microbiomes. An examination was conducted to analyze the microbiota in the feces, and SCFAs were measured using liquid chromatography–mass spectrometry. During Ramadan, researchers found lower amounts of SCFA and beneficial bacteria in the gut. However, after Ramadan, microbial diversity increases, suggesting that the nutrients consumed daily may not be enough to support a healthy gut microbiota [98]. Ali et al. [99] studied 34 healthy adults (18 Pakistanis and 16 Chinese). They found that when people are not geographically isolated, the gut microbiota of people fasting during Ramadan differs significantly from one another, particularly in terms of structure, composition, and alpha and beta diversities. This variation is mostly caused by dietary differences. The beta diversity and frequency of specific signature taxa may have been affected by the intermittent calorie restriction that is associated with Ramadan fasting [99]. In 2022, Su et al. found that fasting during Ramadan changed the composition of the gut microbiota in BALB/c mice (*p* < 0.01) and significantly increased the levels of butyrate-producing Lachnospiraceae and Ruminococcaceae (*p* < 0.01), similar to the outcome in humans [100]. Firmicutes were found in higher abundance in the gut microbiota before fasting, but their levels dropped dramatically by the conclusion of Ramadan fasting, according to a study done in Turkey with 12 healthy adults (*p* < 0.05). At the conclusion of Ramadan, the prevalence of proteobacteria showed a considerable rise (*p* < 0.05). Blautia, Coprococcus, Dorea, Fusicatenibacter, Faecalicatena, Lachnoclostridium, and Mediterraneibacter were among the seven genera whose levels were significantly lower when subjects fasted. The bacterial genera Shigella and Escherichia, on the other hand, saw their populations grow once the fasting month ended. Three cases were identified where there was a negative correlation with food consumption: (1) Ihubacter and protein (rho = −0.54, *p* = 0.0068), (2) Fusicatenibacter and vegetables (rho = −0.54, *p* = 0.0042), and (3) Intestinibacter and nuts (rho = −0.54, *p* = 0.0065). The results suggest that people’s meal choices during Ramadan can affect gut flora, even while fasting for the same amount of time every day [101]. In Su et al. 2021 [102], thirty healthy individuals were included in the study conducted in China. During Ramadan, a 30-day fast was observed, during which time fasting meant going without food and drink for over 16 hours every day. Significant changes in the gut flora are induced by fasting during Ramadan, according to the study. A clear biological rationale for the health benefits associated with intermittent fasting is the rise in butyric acid-producing Lachnospiraceae that occurs as a result of this dietary regimen. Researchers in Turkey found that taxa like Butyricicoccus, Bacteroides, Dialister, Faecalibacterium, Allobaculum, Roseburia, Eubacterium, and Erysipelotrichi were much more abundant in stool samples taken after Ramadan ended, compared to samples taken the day before and the last day of the fast. Butyricoccus pullicaecorum was the most heavily impacted bacterial species by Ramadan fasting, according to random forest analysis. Fasting during Ramadan alters the composition of intestinal flora, according to researchers [103]. The Selen et al. 2024 [104] study conducted in Turkey with 10 healthy participants revealed a notable increase in both alpha and beta diversity of the gut microbiota during Ramadan fasting. The Clostridia class Firmicutes phylum, Clostridiales order, and Ruminococcaceae family exhibited notable declines, whereas the Bacteroidetes Proteobacteria phyla, Alphaproteobacteria, Bacteroidia, Bacteroidales, and Erysipelotrichi classes, Erysipelotrichales, and Actinomycetales orders, Erysipelotrichaceae family, and Prevotella genus showed significant increases. Ramadan fasting alters gut microbiota and improves blood lipid profiles and FABP4 concentrations [104]. Additional investigations with larger sample sizes are necessary to examine the influence of Ramadan on gut microbiota.

### 8.14. Ramadan Fasting and Oxidative Stress

A study In Jordan [105], including fifty healthy individuals, found that increased body weight is associated with augmented oxidative stress and lipid peroxidation and with the effects of Ramadan fasting on oxidative stress influenced by variations in body weight. A research investigation conducted in Turkey with fifty-seven healthy participants showed that Ramadan fasting markedly elevated total antioxidant capacity while diminishing oxidative stress index and total oxidant status [106].

A study by Al-Shafei et al. [107] in Egypt comprised eighty individuals (40 with diabetes and 40 without). Fasting throughout Ramadan markedly increased blood glutathione concentrations in both cohorts. In Kasap et al. [108], a study conducted in Turkey with a hundred healthy pregnant females (50 fasting) revealed that Ramadan fasting significantly decreased the total antioxidant status in the fasting cohort, while no notable differences were observed between the groups regarding total oxidant status and oxidative stress index. A different study by Al-Shafei et al. [109] conducted in Egypt involving forty hypertensive individuals indicated that Ramadan fasting increased glutathione levels, improved blood pressure and lipid profiles, and reduced oxidative stress in patients with hypertension. A study conducted in Turkey [110] with seventy-two pregnant women indicated that maternal fasting during Ramadan in the second trimester does not produce notable impact on maternal oxidative stress. Research in Saudi Arabia [111] with sixty-two healthy women indicated that Ramadan fasting was linked to heightened levels of oxidative stress markers in both groups. A study conducted in Iran [112] with twenty-seven women suffering from polycystic ovarian syndrome showed that fasting during Ramadan produced a notable increase in plasma concentrations of nitric oxide and glutathione. Mrad et al. [113] study conducted in Tunisia involving 15 male patients with chronic obstructive pulmonary disease, indicated that Ramadan fasting did not result in notable alterations in oxidative stress indicators among these individuals. The Asadi et al. [114] study conducted in Iran with twenty-one patients suffering from cerebrovascular issues, coronary artery disease, or peripheral arterial disorders found that fasting during Ramadan significantly lowered plasma concentrations of protein carbonyl groups serum and amyloid A. Ramadan fasting has the potential to diminish oxidative stress and may enhance MASLD and endothelial function.

### 8.15. Ramadan Fasting and Mitophagy

Mitophagy is a selective form of autophagy responsible for the recognition and removal of damaged or dysfunctional mitochondria. By maintaining mitochondrial quality, mitophagy contributes to cellular energy homeostasis, limits excessive production of reactive oxygen species (ROS), and supports mitochondrial adaptation to metabolic stress [115,116]. These processes are particularly relevant to MASLD because mitochondrial dysfunction is implicated in the transition from hepatic steatosis toward inflammation, hepatocellular injury, and fibrosis [117,118]. Experimental and emerging clinical evidence suggests that impaired mitochondrial quality control and altered mitophagy may accompany MASLD and liver fibrosis [117,119]. The potential relevance of mitophagy to MASLD can be considered in the context of hepatic lipid overload. Insulin resistance and increased delivery of fatty acids to the liver increase mitochondrial metabolic demand. Persistent lipid excess can promote mitochondrial ROS production, oxidative damage, impaired mitochondrial respiration, and cellular stress [117,118]. Inadequate removal of damaged mitochondria may further amplify oxidative stress and inflammatory signaling, thereby creating a potential feed-forward mechanism linking metabolic dysfunction to hepatic injury [117,119]. Thus, restoration of appropriate mitochondrial quality control represents a biologically plausible mechanism through which fasting could influence hepatic metabolic health.

Fasting may activate several nutrient-sensing pathways involved in autophagy and mitochondrial quality control. During periods of reduced nutrient availability, activation of AMP-activated protein kinase (AMPK) and sirtuin-1 (SIRT1), together with inhibition of mammalian target of rapamycin (mTOR), can favor autophagic processes and metabolic adaptation [115,116]. Fasting-associated increases in fatty-acid oxidation and ketogenesis may also alter mitochondrial substrate utilization [120,121]. These mechanisms provide a plausible biological basis for enhanced mitochondrial turnover during fasting. However, most detailed evidence linking fasting-induced mitophagy to improved hepatic outcomes has been obtained from experimental or mechanistic studies, and direct evidence demonstrating increased hepatic mitophagy in humans observing Ramadan remains limited [119,120,121]. Evidence specifically related to Ramadan fasting is beginning to emerge. A prospective UAE study involving 51 overweight and obese individuals reported the increased expression of several autophagy-related genes, including LC3B, LAMP2, ATG4D, and ATG5, following Ramadan fasting [122,123]. These findings support an association between Ramadan fasting and activation of autophagy-related pathways, but they do not by themselves establish increased hepatic mitophagic flux or demonstrate improvement in MASLD. Similarly, mechanistic reviews have proposed that intermittent fasting may normalize autophagy and potentially provide hepatic protection in metabolic dysfunction-associated fatty liver disease [119,120,121]. These observations should therefore be interpreted as supportive rather than definitive evidence for a Ramadan-induced mitophagy-mediated hepatic benefit. An additional potential connection involves the gut microbiota. Microbial metabolites, particularly short-chain fatty acids such as butyrate, may influence mitochondrial function and autophagy-related signaling, whereas dysbiosis and endotoxin exposure may promote mitochondrial oxidative stress and impair mitochondrial quality control [122,123,124]. Thus, changes in gut microbial composition during Ramadan could theoretically influence mitochondrial homeostasis. Nevertheless, a direct causal gut microbiota–mitophagy pathway in humans during Ramadan has not yet been established. Overall, mitophagy represents a plausible mechanistic link between fasting-induced metabolic adaptation and mitochondrial health in MASLD. However, the current evidence supports a hypothesis-generating framework rather than a demonstrated clinical mechanism. Future Ramadan studies should directly assess mitochondrial function and mitophagic flux, together with hepatic fat and fibrosis measures, to determine whether changes in mitophagy mediate clinically meaningful hepatic effects [119,120,121].

### 8.16. Ramadan Fasting and Circadian Rhythm

Ramadan fasting should not be considered synonymous with generic intermittent fasting or time-restricted eating. Although all involve periods of food restriction, Ramadan has a distinctive behavioral and temporal pattern characterized by complete abstinence from food and fluid during daylight hours, food consumption predominantly after sunset and before dawn, and frequently altered sleep–wake patterns. The metabolic effects observed during Ramadan may therefore reflect the combined influence of fasting duration, altered meal timing, changes in meal composition and caloric intake, sleep disruption or redistribution, physical activity, and nocturnal light exposure. Consequently, an observed association between Ramadan observance and a metabolic outcome cannot necessarily be attributed to fasting alone. This distinction is particularly important when interpreting studies of MASLD, endothelial function, gut microbiota, and circadian biology. Some metabolic changes may result primarily from reduced energy intake or weight loss, whereas others may arise from the timing of food consumption or changes in sleep and activity. Ramadan therefore represents a complex physiological model combining prolonged daytime fasting with nocturnal feeding and behavioral circadian changes rather than a simple form of conventional time-restricted eating. Ramadan fasting involves changes in sleep habits, meal time, physical activity, and exposure to nocturnal light. Therefore, Ramadan fasting affects circadian biology via fasting–feeding cycles and behavioral alterations [120,121].

Meal timing is particularly important because peripheral clocks are strongly influenced by feeding cues. During Ramadan, the usual daytime feeding pattern is shifted toward the biological night, with substantial energy intake occurring at Iftar and, in many individuals, again at Suhoor. This temporal redistribution of nutrient availability may influence hepatic clock-controlled pathways involved in glucose production, glycogen storage, fatty-acid oxidation, de novo lipogenesis, cholesterol metabolism, and bile acid homeostasis [125,126,127]. Nutrient-sensing pathways provide an important molecular interface between feeding behavior and the circadian clock. AMPK responds to the cellular energy status, whereas SIRT1 and mTOR integrate nutrient availability with transcriptional and metabolic programs. These pathways interact with core clock components and may thereby influence the timing and magnitude of hepatic metabolic responses [122,123,128,129]. However, Ramadan-related circadian effects are not determined by fasting alone. Delayed sleep, fragmented sleep, nocturnal light exposure, and changes in physical activity can alter circadian synchronization and may counteract or modify the metabolic effects of daytime fasting [130,131,132]. Thus, the metabolic consequences of Ramadan may depend on the balance between prolonged daytime fasting and the extent of nocturnal behavioral disruption. Recent studies of time-restricted eating also suggest that the timing of food intake, independent of weight loss, may influence insulin sensitivity, metabolic gene expression, and circadian alignment; however, these findings cannot be directly extrapolated to Ramadan because Ramadan combines daytime fasting with nocturnal feeding and changes in sleep–wake behavior [133,134].

The liver is a major peripheral circadian organ, and disruption of hepatic clock regulation has been associated with abnormalities in glucose metabolism, lipid handling, fatty-acid oxidation, bile acid metabolism, and inflammatory pathways relevant to MASLD [126,128,135]. Recent work further indicates that circadian misalignment may interact with metabolic dysfunction-associated steatotic liver disease through altered hepatic lipid metabolism, inflammation, mitochondrial function, and gut–liver signaling [135,136,137]. Therefore, one plausible hypothesis is that the timing of fasting and feeding during Ramadan could modify hepatic metabolic rhythms through nutrient-sensitive clock pathways. Whether this produces sustained improvement in hepatic steatosis or fibrosis in humans remains uncertain and requires prospective investigation using objective measures of meal timing, sleep, circadian phase, liver fat, and fibrosis.

The interplay between circadian rhythms and hepatic metabolism is especially pertinent to MASLD. The liver has one of the most resilient peripheral clocks, governing daily fluctuations in glucose generation, glycogen storage, cholesterol synthesis, fatty-acid oxidation, de novo lipogenesis, and bile acid metabolism. Circadian misalignment impairs metabolic pathways, fostering insulin resistance, hepatic fat buildup, oxidative stress, and inflammatory signaling, therefore expediting the onset and advancement of MASLD. Conversely, well-timed fasting may enhance metabolic flexibility by aligning hepatic metabolism with circadian rhythms and improving food sensing [9].

A pilot investigation by Anwardeen et al. in Qatar involving 12 female students, including five with normal sleep and seven with disrupted sleep, revealed that fasting individuals with normal sleep patterns demonstrated a reduced HOMA-IR in reaction to physical activity relative to subjects with disrupted sleep. Furthermore, they exhibited superior lipid utilization during exercise, shown by decreased diacylglycerol concentrations, potentially improving sensitivity of insulin and decreasing the probability of type 2 diabetes. Conversely, fasting individuals exhibiting irregular sleep patterns encountered metabolic strain, characterized by the considerable reduction in polyunsaturated fatty acids (PUFAs), monounsaturated fatty acids (MUFAs), and plasmalogens due to physical exertion. These alterations were linked to heightened inflammation and oxidative stress, which may result in metabolic dysregulation [124]. Given the very small sample size, female-only population, and exploratory nature of the study, these findings should be considered hypothesis-generating rather than evidence of a generalizable effect of Ramadan-related circadian disruption on metabolic health.

Al-Rawi et al. in UAE including fifty-seven overweight and obese individuals, comprising forty males and seventeen females, demonstrated that at the conclusion of Ramadan, blood concentrations of melatonin, ghrelin, and leptin considerably (*p* < 0.001) decreased, although salivary cortisol levels remained unchanged in comparison to pre-fasting measurements [138].

Circadian rhythms also govern autophagy and mitochondrial quality regulation. Autophagic activity has a diurnal rhythm regulated by clock genes and nutrient-responsive signaling pathways. Fasting activates AMPK and SIRT1, inhibits mTOR, accelerates autophagy and mitophagy, promotes the elimination of damaged proteins and defective mitochondria, and decreases oxidative stress. Ramadan fasting, which integrates extended fasting with daily cycles of fasting and eating, may enhance the circadian control of autophagy, thereby promoting enhanced cellular homeostasis. While mechanistic data from animal models robustly supports this connection, actual human investigations during Ramadan are still limited [123].

There is a bidirectional link between circadian rhythms and the gut microbiota. The gut microbiome displays daily fluctuations in microbial composition, metabolite synthesis, and gene expression, mostly influenced by dietary habits. Changes in meal time during Ramadan may modify microbial rhythmicity, influencing the synthesis of short-chain fatty acids, bile acid metabolism, intestinal permeability, and immunological modulation. Microbial metabolites subsequently modulate peripheral clock gene expression in the liver and intestine, potentially creating a reciprocal gut–circadian interaction that could influence hepatic metabolism and endothelial function. However, the directionality and clinical significance of this interaction during Ramadan remain incompletely defined [139].

Collectively, available evidence suggests that Ramadan fasting may influence insulin sensitivity, oxidative stress, inflammation, endothelial-related biomarkers, gut microbial composition, mitochondrial quality-control pathways, and circadian metabolic regulation. However, whether these changes translate into clinically meaningful improvement in MASLD or prevention of its progression remains uncertain. The available evidence is predominantly based on short-term studies, relatively small cohorts, experimental models, or indirect biomarkers, and therefore does not establish a causal effect of Ramadan fasting on the long-term progression of MASLD.

### 8.17. Study Limitations

Several limitations should be considered when interpreting the findings of this narrative review. First, the review was designed as a narrative synthesis rather than a prospectively registered systematic or scoping review. Although PubMed and Scopus were searched using predefined concepts and eligibility considerations, the search strategy was not designed to provide the exhaustive coverage or formal risk-of-bias assessment characteristic of a systematic review. Consequently, selection and interpretation of the literature may be influenced by the heterogeneity and availability of published evidence. Second, the number of studies directly investigating Ramadan fasting in individuals with MASLD remains limited. The available literature includes observational and retrospective human studies as well as experimental animal studies and research examining intermittent fasting or other fasting-related interventions. These studies differ in population characteristics, disease definitions, fasting protocols, duration, outcomes, and methodological quality, limiting direct comparison and causal inference. Evidence from generic intermittent fasting, time-restricted eating, or experimental fasting should therefore be regarded as indirect or extrapolated evidence rather than as direct evidence of the effects of Ramadan fasting. Third, the proposed gut–mitophagy–circadian axis represents a hypothesis-generating conceptual framework rather than a pathway that has been demonstrated in humans with MASLD undergoing Ramadan fasting. Although individual components of this framework are supported by experimental and observational evidence, there is currently insufficient human evidence demonstrating that Ramadan fasting simultaneously produces coordinated changes in gut microbiota, circadian regulation, mitophagy, endothelial function, and hepatic outcomes. In particular, changes in circulating metabolic markers, liver enzymes, or expression of autophagy-related genes should not be interpreted as direct evidence of increased hepatic or endothelial mitophagy. Fourth, many of the available Ramadan studies are observational and may be affected by residual confounding. Changes in dietary composition, caloric intake, sleep–wake patterns, physical activity, medication adherence, hydration, and other lifestyle behaviors during Ramadan may independently influence metabolic and hepatic outcomes. Therefore, observed associations cannot necessarily be attributed solely to fasting. Finally, most available studies evaluate short-term surrogate outcomes, such as body weight, insulin resistance, lipid concentrations, liver enzymes, inflammatory markers, or hepatic steatosis. Improvements in these parameters should not be interpreted as evidence that Ramadan fasting prevents progression to advanced fibrosis, cirrhosis, hepatocellular carcinoma, or major cardiovascular or hepatic clinical events. Similarly, experimental evidence suggesting antitumor effects of fasting cannot currently be extrapolated to Ramadan fasting or used to recommend fasting as an anticancer intervention.

## 9. Conclusions

**Figure 3:**
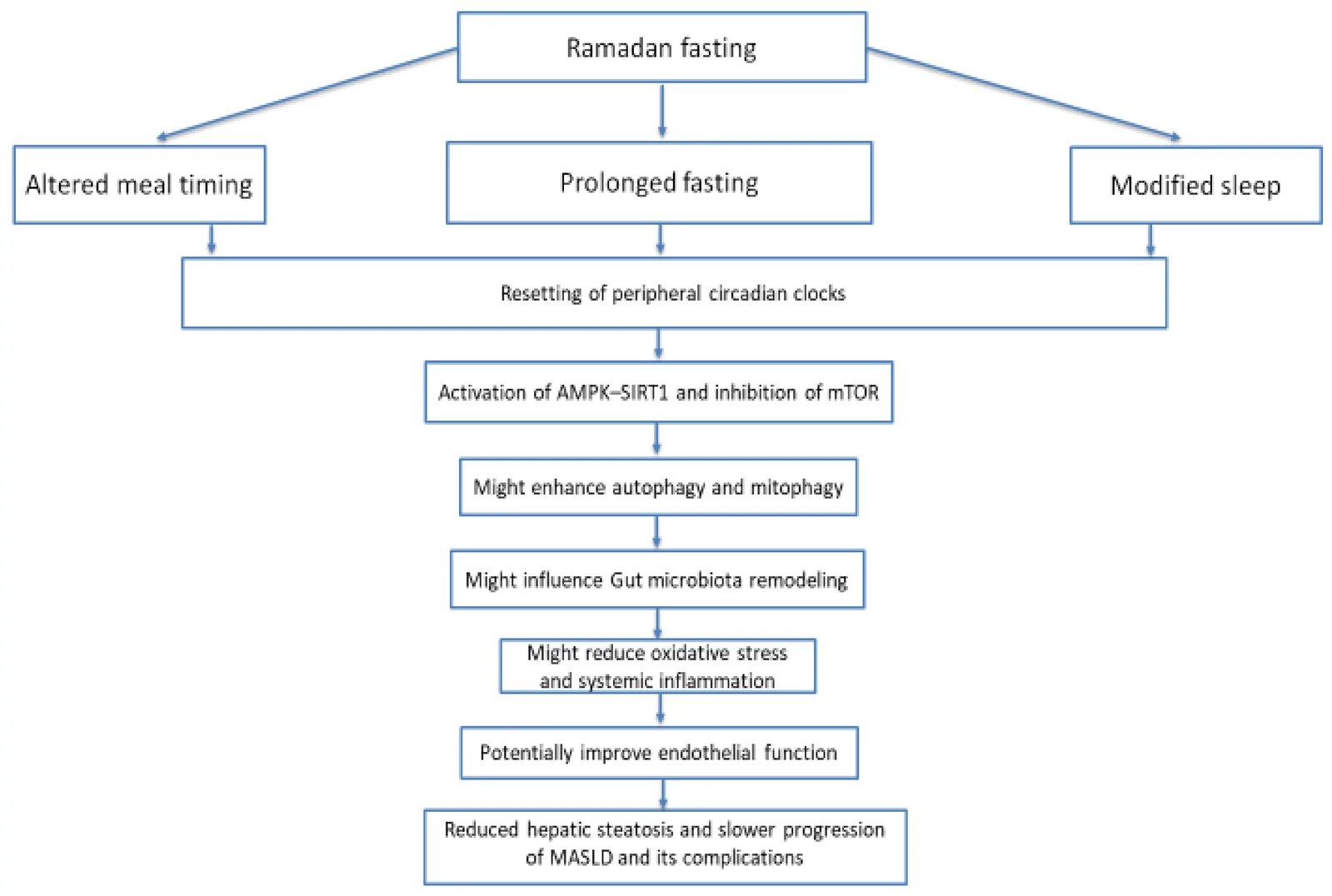
The relationship between Ramadan fasting, circadian rhythm, mitophagy, gut microbiota, endothelial function and hepatic health.

MASLD and its associated endothelial dysfunction arise from interconnected metabolic, inflammatory, oxidative, vascular, intestinal, mitochondrial, and circadian mechanisms. Available evidence suggests that Ramadan fasting may favorably influence several of these factors, including body weight, insulin sensitivity, oxidative stress, inflammation, endothelial-related biomarkers, and gut microbial composition. Emerging evidence also suggests that fasting may influence autophagy and mitochondrial quality-control pathways and interact with circadian metabolic regulation (Figure 3).

Nevertheless, the evidence should be interpreted cautiously. Ramadan fasting is a complex physiological and behavioral intervention that combines prolonged daytime fasting with nocturnal feeding, altered meal timing, changes in sleep–wake patterns, physical activity, and nocturnal light exposure. Therefore, observed metabolic effects cannot always be attributed to fasting itself. In addition, direct human evidence linking Ramadan fasting with hepatic mitophagy, circadian reprogramming, or a coordinated gut–mitophagy–circadian pathway remains limited.

The gut–mitophagy–circadian axis proposed in this review should consequently be considered a mechanistic hypothesis that integrates currently available evidence rather than an established causal pathway. Larger prospective, multicenter, and randomized studies are needed to determine the long-term hepatic, vascular, microbiome, circadian, and mitochondrial effects of Ramadan fasting in individuals with MASLD. Future studies incorporating quantitative liver imaging, fibrosis assessment, longitudinal microbiome profiling, circadian biomarkers, and direct markers of mitochondrial quality control and mitophagy may help determine whether these interconnected pathways mediate clinically meaningful hepatic benefits.

Figure 3 presents a proposed conceptual model rather than a demonstrated causal pathway. The model integrates several biological processes that may be influenced by Ramadan fasting, including altered meal timing, gut microbial composition and metabolites, nutrient-sensing pathways, circadian signaling, autophagy/mitophagy, oxidative stress, endothelial function, and hepatic metabolism. The arrows linking these processes represent proposed or biologically plausible interactions and should not be interpreted as evidence of a directly demonstrated causal sequence in patients with MASLD. Evidence supporting individual components of the model comes from different populations and experimental settings. Therefore, the model is intended to generate testable hypotheses concerning whether Ramadan fasting can simultaneously influence gut microbial rhythmicity, mitochondrial quality control, circadian regulation, endothelial function, and hepatic metabolic pathways.

## Figures and Tables

**Table 1:** Overview of studies investigating Ramadan fasting or fasting-related interventions in MASLD/NAFLD.

Study	Study Design	Population/ Sample Size	Diagnostic Criteria/Disease Definition	Fasting or Intervention	Duration/ Follow-Up	Main Endpoints	Principal Findings	Main Limitations	Evidence Category
Alasmari et al. (2024) [12]	Experimental animal study	Rats with experimentally induced NAFLD	Experimentally induced NAFLD	Ramadan fasting model	Ramadan-like fasting intervention; duration as reported in the original study	Body weight, lipid profile, ALT, AST and hepatic outcomes	Reduced body weight, cholesterol, triglycerides, LDL, ALT and AST; findings suggested hepatoprotective and metabolic effects	Animal model; findings cannot be directly extrapolated to humans or to patients with MASLD undertaking Ramadan fasting	Mechanistic/indirect
Mari et al. (2021) [79]	Retrospective case–control study	Patients with fatty liver disease; Ramadan-fasting and comparison groups	Fatty liver disease assessed according to the study’s clinical/imaging criteria	Ramadan fasting	Ramadan month; retrospective assessment	Body weight, insulin sensitivity, inflammatory markers and indicators of fatty liver/NASH severity	Ramadan fasting was associated with improvements in body weight, insulin sensitivity, inflammatory indicators and markers related to fatty liver disease severity	Retrospective design; potential confounding from diet, physical activity and other Ramadan-related lifestyle changes; limited ability to establish causality	Direct/clinical, but observational
Ebrahimi et al. (2020) [80]	Clinical observational study	Patients with NAFLD	NAFLD diagnosed according to study criteria	Ramadan fasting	Approximately one Ramadan month	Liver function tests, total cholesterol, atherogenic index of plasma and visceral adiposity index	Ramadan fasting was associated with improved liver function and reduced total cholesterol, with reductions in atherogenic and visceral adiposity indices	Observational design; limited sample size and follow-up; cannot establish long-term effects on fibrosis or clinical outcomes	Direct/clinical, but observational
Badran et al. (2022) [81]	Interventional/observational study of intermittent fasting	Patients with NAFLD	NAFLD diagnosed according to study criteria	Intermittent fasting; not specifically Ramadan fasting	Study intervention period as reported in the original publication	Laboratory, anthropometric and radiological parameters	Improvements were reported in anthropometric, biochemical and ultrasonographic parameters, particularly in selected patient subgroups	Not a Ramadan-specific intervention; therefore, findings should not be presented as direct evidence for Ramadan fasting; imaging does not establish improvement in fibrosis or long-term outcomes	Indirect/extrapolated
Aliasghari et al. (2017) [82]	Observational trial	Patients with NAFLD	NAFLD diagnosed according to study criteria	Ramadan fasting	One Ramadan month	Body composition, blood pressure, glucose metabolism, insulin and inflammatory markers	Improvements were reported in body measures, insulin-related parameters, fasting blood glucose and inflammatory markers	Observational design; possible dietary, sleep and activity-related confounding; limited information on long-term hepatic outcomes	Direct/clinical, but observational
Alam et al. (2019) [83]	Clinical observational study	Patients with lean NASH	Histologically assessed NASH	Weight-reduction intervention; not a Ramadan-fasting study	As reported in the original study	Histological activity and fibrosis	Weight reduction improved several aspects of histological activity in lean NASH	Not a Ramadan study and therefore cannot establish an effect of Ramadan fasting; indirect evidence only	Indirect/extrapolated
Ahmed & Ahmed (2024) [11]	Narrative review	Evidence concerning Ramadan fasting and MASLD	Review of MASLD-related literature	Ramadan fasting	Not applicable	Metabolic and hepatic risk factors	Proposed that Ramadan fasting may improve MASLD-related risk factors, particularly through weight loss and metabolic effects	Narrative review; conclusions depend on heterogeneous primary studies; does not provide independent clinical evidence	Secondary/contextual evidence

## Data Availability

No new data were created or analyzed in this study. Data sharing is not applicable to this article.
